# Nutritional needs of children with disabilities in the UAE: understanding predictors and mediators of nutritional knowledge and practices

**DOI:** 10.1186/s40795-022-00605-9

**Published:** 2022-10-04

**Authors:** Maxwell Peprah Opoku, Ashraf Moustafa, Noora Anwahi, Hala Elhoweris, Fatima Alkatheeri, Najwa Alhosani, Anwar Alameri, Shashidhar Belbase

**Affiliations:** 1 Special Education Department, United Arab Emirates University, Al-Ain, United Arab Emirates; 2 Curriculum and Method of Instruction United Arab Emirates University, Al-Ain, United Arab Emirates

**Keywords:** Nutrition, Children with disabilities, Parents, Teachers, Health literacy, UAE

## Abstract

**Background:**

There is a high estimated prevalence of obesity and poor eating habits among children with disabilities. Unfortunately, the extent of parental and teachers’ awareness of the dietary needs and nutritional requirements of children with disabilities has been understudied. This study aims to explore the predictors and mediators of nutritional knowledge and practices among parents and teachers of children with disabilities in the United Arab Emirates (UAE) using Nutbeam’s hierarchical health literacy model as a framework to test three hypotheses.

**Methods:**

A total of 149 parents and teachers were recruited from rehabilitation centres in two of the seven Emirates in the UAE. The revised Food and Nutritional Literacy Scale (FNLIT) was used for data collection. The revised scale was validated and its reliability was assessed using SPSS and AMOS version 28 to compute exploratory factor analysis and structural equation modelling (confirmatory factor analysis and path analysis), respectively.

**Results:**

The results confirmed a four-factor structure for FNLIT, and all three hypotheses were supported by the study findings. We confirmed a relationship between understanding and practical nutritional skills (Hypothesis I), and found that two practical nutritional skills, functional and interactive, combined to predict the understanding of nutritional needs of children with disabilities (Hypothesis II). Hypothesis III was partially supported in that participant type mediated the relationship between functional, interactive, and understanding factors. The convergent and discriminant validities of the scale were confirmed, and path analysis showed the ability of practical skills to predict knowledge.

**Conclusion:**

The study concludes on the need for public education on nutritional needs as well as developing the capacity of teachers and parents to implement appropriate eating programmes for children with disabilities.

## Background

Nutrition is a key factor in the physical and cognitive development of children and may impact their educational performance [1–5]. Children need optimal access to food to enhance their development regardless of race, religion, and ethnicity. Specifically, children have the right to have good health, clean water, healthy food, and a good environment according to the United Nations Convention on the Rights of the Child [6]. Consequently, international organisations such as UNICEF, FAO, and the UN, through their Sustainable Development Goals, under zero hunger (Goal 2), have reiterated the need for countries to promote access to nutritional food for all [7]. Indeed, the FAO [8–10] recommended the need for countries to have nutritional indicators to gather data on the quality of the foods children consume and to maintain an international database. Although attention has been paid to issues regarding children’s overall access to quality food and nutrition [11], discussions on the nutritional needs of children with disabilities are rare, scarce, and uncoordinated. Millions of healthy children with disabilities remain undernourished worldwide [12].

In this study, disabilities refer to impairments, including sensory, physical, and cognitive, that impede individuals’ daily living experiences and can affect their ability to communicate, perform daily life functions, and engage in social and emotional interactions [13, 14]. According to the World Health Organization [14], at least 10% of the global population is living with a form of disability and 2–4% with severe disability, requiring attention to be paid to their needs and development. The recent Convention on the Rights of Persons with Disabilities is a key international instrument aimed at enhancing the living conditions of persons with disabilities [15]. For instance, Article 25 espouses the health needs of individuals with disabilities and encourages countries to make food and other nutritional needs accessible to them [15]. Although some progress has been made in terms of optimising the development of children with disabilities, formidable barriers still exist when it comes to accessing social services such as healthcare and education [14, 16, 17]. There are ongoing discussions on ways to enhance the inclusion of people with disabilities in society, but ways to enhance the eating habits of children with disabilities have received little traction [14–17].

The two most important individuals in the lives of children with disabilities are teachers and parents [17–20]; it is undeniable that when these children are of school-going age, they spend more time with either their teachers or parents than with any other professional. Indeed, in discussions on the nutrition and health needs of children with disabilities, parents and teachers are key stakeholders in documenting their knowledge and practices. This research strongly indicates that both teachers and parents contribute enormously to the development of the well-being of children with disabilities [20, 21], and a large body of literature has documented the daily struggles of these parents, [22–28]. This encompasses issues pertaining to challenges in behaviour management, raising children, and facilitating socialisation in society. Additionally, teachers, including special educators, face challenges such as behaviour management, lack of teaching resources, and unfriendly teaching environments for teaching children with disabilities [29–32]. It is useful to state that some parents and teachers have succeeded in promoting their development. However, there is still an urgent need for solutions to the myriad challenges that are encountered.

Previous studies have attempted to explore the eating habits of children with disabilities. In several countries, disability is nearly synonymous with poor eating habits. For example, a large body of literature reports a strong association between disability and obesity [16, 33–37]. Specifically, children with disabilities have been reported to be more likely overweight or underweight [16], which has been linked to poor diet, irregular eating patterns, lack of exercise, sleep disorders, and other genetic factors [33]. Other studies conducted in countries, such as Iran have, reported populations with limited knowledge of their health needs [38]. Although these studies are insightful for developing some understanding of the issues, such scholarly discussions have received limited attention in non-Western settings, such as the United Arab Emirates (UAE). The federal government of the UAE has developed a nutritional access strategy to promote access to food for all children [39]; however, there are no measurement indicators to assess the knowledge of stakeholders, such as teachers and parents, regarding the food and nutritional needs of children with disabilities. Furthermore, to the best of our knowledge, no researcher has attempted to understand the knowledge of parents and teachers regarding the nutritional needs of children with disabilities in the UAE. Thus, this study aims to assess the structural validity and reliability of a nutritional measure and to understand the predictors of nutritional awareness among teachers and parents in the UAE.

## Theoretical framework

Nutbeam’s hierarchical health literacy model guided this study [40–45]. We defined health literacy as an individual’s level of knowledge, cognitive and social skills, capacity to understand health information, and capacity to make decisions aimed at improving their health and well-being [40]. Nutbeam conceptualised health literacy as an asset [41, 42] but argued that developing individuals’ knowledge is not sufficient. Rather, it is vital to empower individuals to control their health needs [43, 44]. Individuals such as parents and teachers who have received health education should be trained to assert their health rights and those of others in their sphere of control [42, 45]. A well-trained individual is in a better position to engage in a given activity, but health literacy training without building confidence will lead to non-application of health knowledge. Thus, in the UAE, individuals such as teachers and parents need to be educated and empowered to apply nutritional knowledge to expand the access of children with disabilities to high-quality food and nutrition.

For this study, based on Nutbeam’s health literacy model, we proposed a relationship between health knowledge and practical skills (Hypothesis I), and that practical nutritional skills would combine to predict nutrition literacy (Hypothesis II). Given the intricate connection between one’s training and knowledge [46], we also hypothesised that being a teacher or parent of a child with disabilities could affect one’s nutritional knowledge and practical application skills (Hypothesis III). The model guided the development of the Food and Nutritional Literacy Scale (FNLIT) (47–50), which has two broad domains: knowledge and skills. This scale was found to be valid and was supported by a solid theoretical foundation. Thus, in this study, we adopted the FNLIT in the context of UAE to study parents’ and teachers’ awareness of the food and nutritional needs of children with disabilities.

## Overview of the study

The UAE is a federation of seven sheikdoms, namely Abu Dhabi, Ajman, Dubai, Fujairah, Ras Al Khaimah, Sharjah, and Umm Al-Quwain. The country has been described as the centre of human excellence in human development. As part of its efforts to achieve the UN Sustainable Development Goals, the government has implemented several initiatives aimed at promoting the development of citizenry. One such policy is to develop a national food and nutrition strategy [39, 51]. The proposed policy comes with several nutrition guidelines for children living in the country, and even identifies parents and teachers as important stakeholders in promoting healthy eating habits among children. Although the development of policy is believed to be a positive step, there is little information about whether teachers and parents can play the role of policy documents assigned to them. The policy makes a brief reference to the nutritional well-being of all age groups, including people with special needs, but it does not clearly mention the needs of children with disabilities who have been consistently found to be at risk of vulnerability in the context of UAE [52–55].

Overall, the study was guided by the following research questions:

1. Is there a relationship between nutritional knowledge and practical skills among teachers and parents of children with disabilities in the UAE?
2. Do teachers’ and parents’ practical skills in nutrition combine to predict their understanding of the nutritional needs of children with disabilities in the UAE?
3. Does being a parent or teacher moderate the relationship between practical skills and the understanding of the nutritional needs of children with disabilities in the UAE?

## Methods

The study reported here was guided by a cross-sectional design as it attempted to capture the views of a section of the population at a point in time. The design enabled an understanding of the perspectives of parents and teachers regarding the nutritional needs of children with disabilities at a given time [56].

## Study participants

The fundamental role of parents and teachers contributed to their consideration for this study. To the best of our knowledge, both groups spent more time with students with disabilities than with any other stakeholder group. In this study, the participants were drawn from two main areas, the Emirates of Abu Dhabi and Dubai, which we selected because they are the major hubs in the UAE for rehabilitation. Both probability and non-probability sampling strategies were used to recruit participants. We used simple random and snowball sampling to recruit the participants. Specifically, we contacted a number of local rehabilitation centres in an attempt to reach potentially interested parents and special education teachers, but we also directly contacted individual teachers and parents who were known to the research team or others. The recruitment of study participants was guided by the following inclusion criteria: (a) involvement in the development of children with disabilities, (b) ability to read either Arabic or English, and (c) consent to participate in this study. In effect, persons who were not involved in the development of children with disabilities, were unable to read Arabic and English and lacked the capacity to consent were excluded from this study.

Using online sample size calculator (http://www.raosoft.com/samplesize.html?nosurvey), the expected sample for this study was 138. The confidence interval was set at 95%, 5% margin error and response distribution set at 90%. Table 1 presents a summary of the 149 participants of this study. Notably, the sample was fairly equally divided between special education teachers (52%) and parents of children with disabilities (48%). Additionally, 62% were Emiratis, compared to 38% who were expats.

## Instrument

A two-part instrument was used for the data collection. The first part collected data on the demographic characteristics (participant type, nationality, age, sex, educational qualification, awareness of nutritional policy, and training in nutrition) of the study participants, which were selected based on a review of the literature [33–37, 57] and suggestions from experts who reviewed the research instrument. The second part of the study survey was conducted with the revised FNLIT [47]. The instrument was originally developed to assess children’s understanding of food and nutrition in Iran, and we revised certain items to make them more suitable for this study. For instance, the items were reworded so that they could measure teachers’ and parents’ understanding of the nutritional needs of children with disabilities.

**Table 1:** Participants’ demographic characteristics

Category (N = 149)	Sample (%)
***Participant type***	
Parent Special education teacher	71 (48%) 78 (52%)
***Nationality (n = 146)***	
Emirati Expat	91 (62%) 55 (38%)
***Gender***	
Male Female	26 (17%) 123 (83%)
***Age (n = 146)***	
21–30 years 31–40 years 41 years and above	38 (26%) 59 (40%) 49 (36%)
***Nutritional policy (n = 144)***	
Familiar Never heard	99 (69%) 45 (31%)
***Training in nutrition (n = 143)***	
Taken PD No training	106 (74%) 37 (26%)

PD = professional development

The FNLIT comprises 42 items in two domains: cognition and skills. The cognitive domain comprises two subscales: understanding (n = 10 items) and knowledge (n = 5 items), where knowledge refers to general education and deeper insight into appropriate food for children with disabilities and understanding refers to individuals’ ability to read and interpret nutritional information. The skills domain of the FNLIT comprises four subscales: functional (n = 10 items), interactive (n = 7 items), food choice (n = 6), and critical (n = 4 items); functional connotes individuals’ ability to access and apply nutritional information, interactive connotes the capacity to engage in discussion with others about food and literacy, critical suggests the ability to analyse nutritional information, and food choice refers to decision making concerning food and nutrition for children with disabilities.

The revised FNLIT was reviewed by six experts, two each in the fields of public health, nutrition, and special education, and their feedback was incorporated into the revised draft used for data collection. We also pilot-tested the tool with ten participants, five special education teachers, and five parents of children with disabilities who were outside the scope of the study. The pilot yielded an appropriate reliability score as well as feedback from the participants, which we also incorporated into the final tool.

## Procedure

The study and protocols were approved by the Human Research and Ethics Committee of UAE University (ERS_2021_8430). Subsequently, formal emails were sent to rehabilitation centres and schools to participate. Because of the outbreak of coronavirus disease 2019 (COVID-19), all communication was virtual, and we collected the study survey data using Google Forms. Schools that approved to participate in this study were sent an online link to be forwarded to both teachers and parents whose children were enrolled at the centres; the schools had social media platforms for both teachers and parents, which made it easier for them to share the instrument. We also relied on other informal networks for data collection and contacted parents and special educators who were already known to the research team. The study questionnaire was written in both Arabic and English, and the online platform was open for three months (December 2021–February 2022). Participants were sent an informed consent document containing the study’s objectives, their right to close the questionnaire at any time without consequences, and a guarantee of anonymity. All participants signed a consent form before participating in the study.

### Data analysis

We first transferred the data to Microsoft Excel for cleaning, and then to SPSS version 28 for further analysis. We collected initial data from 151 participants, but identified two who did not complete the survey; thus, we eliminated their responses from the analyses. Little’s missing completely in the random test (58) showed that random missing data accounted for only 5% of the total data, and we performed expectation maximisation to impute the missing values. Next, we performed exploratory factor analysis (EFA) of the data to validate the dimensionality of the FNLIT scale (Table 2). Specifically, we calculated the Kaiser–Meyer–Olkin value, setting the cut-off at 0.6 and calculated Bartlett’s test of sphericity. We set the acceptable loadings for the items at 0.30 [58] and, following the EFA, transferred the data to AMOS version 28 to answer the research questions.

To answer Research Questions 1 and 2, we used structural equation modelling. First, we performed a confirmatory factor analysis (CFA) to understand the validity of the structure [59] that emerged from the EFA using the chi-square goodness-of-fit test and other fit indices: the comparative fit index (CFI), Tucker–Lewis index (TLI), root square error of approximation (RMSEA), standard root mean square residual (SRMR), and regression weight [59–64]. Based on the literature, we established the following cut-offs to indicate good model fit: a chi-square of less than 0.5 [62], CFI and TLI of 0.90 or greater, RMSEA of less than 0.10, and RMSR of less than 0.10 [59–62] as well as a regression weight of at least 0.50 [63]. Due to a lack of consensus among authors on acceptable fit indices, Weston and Gore [60] argued that authors decide on criteria for assessing the appropriateness of models using multiple indices. In this study, the model fitness was determined by satisfying at least three thresholds.

We tested convergent and discriminant validity to examine the reliability of the data, setting a standardised regression weight of at least 0.50, a composite reliability of at least 0.7, and an average variance extracted of at least 0.50 to establish convergent validity and examine discriminant validity using the square roots of the average variance extracted and the estimated correlation coefficients between the latent variables. We checked the correlations between cognitive skills (Research Question 1) against the following criteria: small (below 0.30), medium (0.30–0.49), and large (at least 0.50) [58]. Next, we performed a path analysis to understand whether practical skills would predict cognitive skills (Research Question 2).

To answer Research Question 3, we used AMOS to compute a moderation analysis to determine the influence of participant type on the relationship between cognitive and practical skills. We created two groups for analysis to understand the influence of the mediator on independent and dependent variables. The mediator was participant type, practical skills were the independent variables, and cognitive skills were the outcome variables. In the imputation of the model, we set the bootstrap at 500 and the bias-corrected confidence intervals at 95. Here, we assessed the appropriateness of the model using goodness-of-fit indices and other indices.

## Results

### Validity of the model

An initial inspection of the correlation matrix showed the presence of coefficients of at least 0.30 between the items, the Kaiser–Meyer–Olkin value was 0.87, and Bartlett’s test of sphericity was significant at p = .001. Principal component analysis revealed the presence of four components with eigen values exceeding 1, accounting for 31%, 8%, 6%, and 5% of the variance. Visual inspection of the screen plot revealed four clear breaks, providing an impetus for the retention of the four factors.

**Table 2:** Summary of exploratory factor analysis

Item	Factor I	Factor II	Factor III	Factor IV
Q1	0.54			
Q2	0.57			
Q3		0.30		
Q4	0.62			
Q5	0.76			
Q6	0.73			
Q8	0.60			
Q9		0.44		
Q10	0.44			
Q11		0.31		
Q12		0.70		
Q13		0.69		
Q14		0.44		
Q15		0.72		
Q16	0.37			
Q17		0.34		
Q18		0.40		
Q19	0.57			
Q20		0.31		
Q21		0.51		
Q22		0.34		
Q23		0.48		
Q24		0.31		
Q25		0.33		
Q26			0.44	
Q27			0.55	
Q28			0.58	
Q29			0.56	
Q30			0.51	
Q31				0.34
Q32			0.33	
Q33				0.47
Q34			0.68	
Q35				0.52
Q36			0.47	
Q37				0.44
Q38				0.61
Q39				0.67
Q40				0.71
Q41				0.31
Q42				0.37

Factor I = understanding; Factor II = functional; Factor III = interactive; Factor IV = food choice

The four identified factors contributed 45% of the variance, with the factors making the following individual contributions: Factor 1, 30%; Factor II, 7%; Factor III, 4%; and Factor IV, 3%. Oblimin rotation showed that 41 items yielded a loading of at least 0.30, as follows: Factor I (understanding, n = 9), Factor II (functional, n = 15), Factor III (interactive, n = 8), and Factor IV (food choice, n = 9).

For the CFA to ascertain the structural validity of the FNLIT, we computed three CFA models in which three indicators met the required threshold (chi-square = 2.34, RMSEA = 0.10, and RMSR = 0.10). However, individual checks showed that seven items had factor loadings below 0.50; therefore, we deleted [64] those seven items (Q3, Q9, Q11, Q14, Q32, Q36, and Q42) before running a second model. The decision to delete the items was to enhance the fit indices.

**Table 3:** Summary model fit indices

	df	χ^2^/df	CFI	TLI	RMSEA	SRMSR
Model 1	773	2.34	0.69	0.67	0.10	0.10
Model 2	521	2.45	0.74	0.74	0.10	0.10
Model 3	489	2.47	0.72	0.74	0.05	0.08
Model 4	458	2.41	0.96	0.94	0.06	0.10
Model 5	489	2.64	0.91	0.96	0.06	0.22
Model 6	884	2.04	0.86	0.91	0.08	0.12

CFI = comparative fit index; TLI = Tucker–Lewis index; RMSEA = root square error of approximation; SRMR = standard root mean square residual

**Fig. 1:**
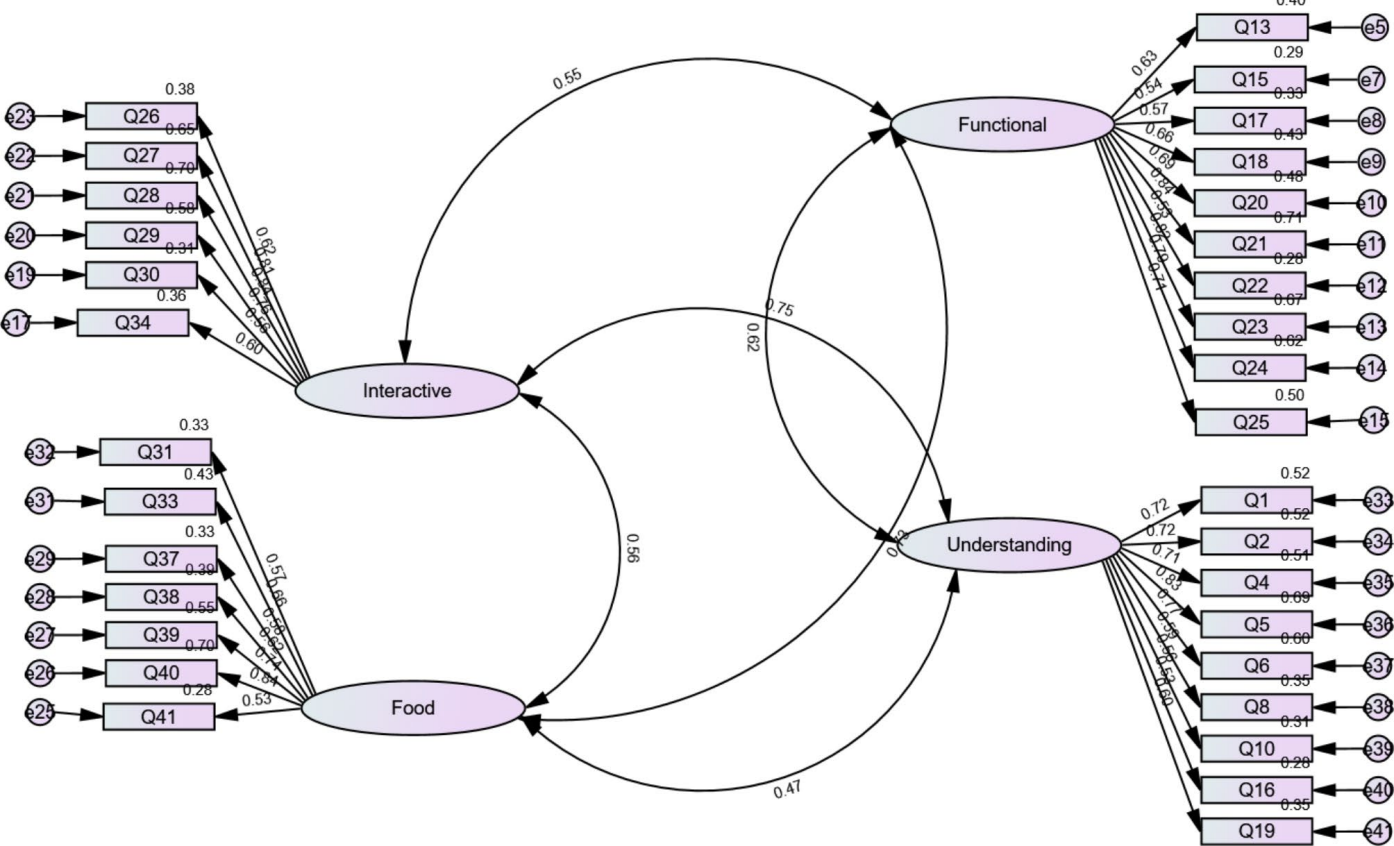
Summary of CFA.

Model two also yielded acceptable fitness indices (chi-square = 2.45, RMSEA = 0.10, RMSR = 0.10), but one item (Q12) had a factor loading less than the acceptable 0.50, resulting in its removal. A third model also had a factor that loaded below 0.50, but finally, the fourth model yielded appropriate factor loadings of above 0.50 and fit indices (chi-square = 2.41, CFI = 0.96, TLI = 0.94, and RMSEA = 0.06) (see Table 3, Model 4, and Fig. 1). CFA confirmed 32 items on the four factors: Factor I (understanding, n = 9 items), Factor II (functional, n = 10), Factor III (interactive, n = 6), and Factor IV (food choice, n = 7).

Next, as noted above, we assessed the reliability of the FNLIT based on convergent and discriminant validity, using the following tests for the former: standardised regression weight, composite reliability, and average variance extracted. First, the factor loadings, as observed using the standardised regression weight, met the required 0.50 thresholds, and the results of the composite reliability for the measures were all at least 0.70 (understanding = 0.88; functional = 0.90, interactive = 0.85, and food choice = 0.84). Additionally, except for food choice, all subscales met the 0.50 cut-off for the average variance extracted.

The discriminant validity findings showed that nearly all the square roots of the average variance extracted exceeded the correlation coefficients of the scales. Cronbach’s alphas were as follows: understanding = 0.88, functional = 0.89, interactive = 0.85, food choice = 0.83. Based on this, we determined that the revised FNLIT was sufficiently reliable to assess the awareness of food and nutrition for children with disabilities in the UAE.

We also identified medium to large correlations among the subscales. Specifically, the relationship between food choice and understanding was medium, the between the other subscales were large, and between food choice and functional (r = .73) was the largest (see Table 4).

## Predictors of cognitive skills

**Fig. 2:**
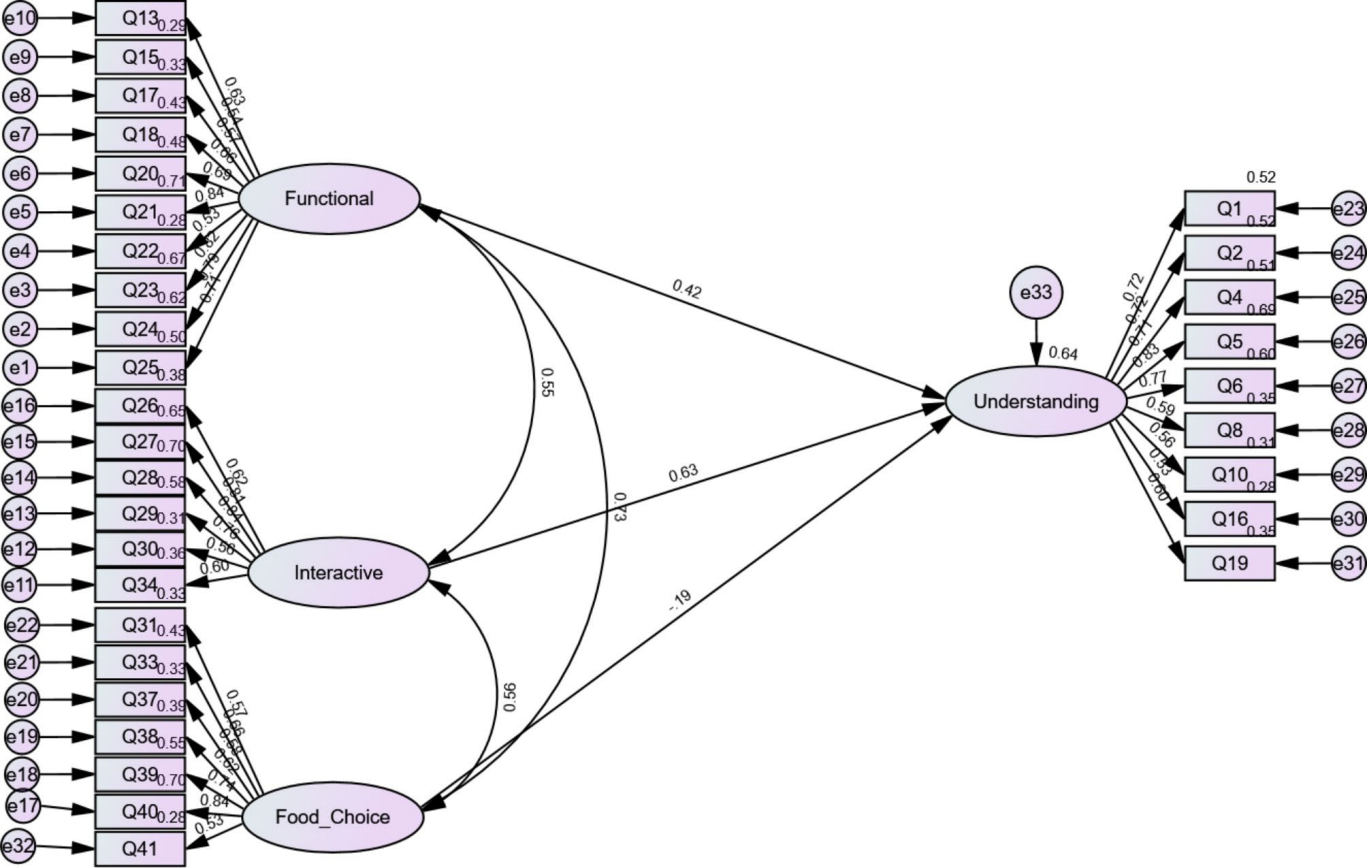
Path analysis of skills predicting cognition

We conducted a path analysis to test the relationship between cognition and nutritional skills. Among the predictors, interactive (b = 0.63, p = .001) and functional (b = 0.42, p = .001) made significant contributions to the variance in understanding, and food choice (b = − 0.19, p = .10) made no contribution (Fig. 2).

**Table 4:** Summary of construct reliability

Construct	Factor loading	CR	AVE	Factor correlation			
				**Understanding**	**Functional**	**Interactive**	**Food choice**
Q1	0.72	0.88	0.5	0.71##			
Q2	0.72						
Q4	0.71						
Q5	0.83						
Q6	0.77						
Q8	0.59						
Q10	0.56						
Q16	0.53						
Q19	0.6						
Q13	0.63	0.9	0.5	0.62#	0.71##		
Q15	0.54						
Q17	0.57						
Q18	0.66						
Q20	0.69						
Q21	0.84						
Q22	0.53						
Q23	0.82						
Q24	0.79						
Q25	0.71						
Q26	0.62	0.85	0.5	0.75#	0.55#	0.71##	
Q27	0.81						
Q28	0.84						
Q29	0.76						
Q30	0.56						
Q34	0.6						
Q31	0.58	0.84	0.43				0.66##
Q33	0.66						
Q37	0.58						
Q38	0.62						
Q39	0.74						
Q40	0.84						
Q41	0.53			0.45#	0.73#	0.56#	

*CR = composite reliability; AVE = average variance extracted; # = correlation coefficient; ## = square root of average variance*

## Mediators of cognition and skills

The mean FNLIT scores for both special education teachers and parents were as follows: understanding, *M* = 3.89, *SD* = 0.66; functional, *M* = 4.25, *SD* = 0.50; interactive, *M* = 3.53, *SD* = 0.68; and food choice, *M* = 3.87, *SD* = 0.57 (see Table 5).

**Table 5:** Summary of means of food and nutritional literacy scale

	Items	M (SD)
	Understanding	3.89 (0.66)
Q1	When shopping for children with disabilities, I pay attention to the nutritional information about food ingredients	3.89 (1.02)
Q2	When shopping for children with disabilities, I pay attention standardized labelling on food packages for you children with disabilities	3.78 (0.99)
Q4	I can easily understand the nutrition facts (e.g. amount of energy, sugar, protein, etc.) on food packages and its health effects to children with disabilities	3.89 (0.99)
Q5	I can easily understand nutritional issues related to children with disabilities I read about in newspapers, magazines, and brochures.	3.82 (0.93)
Q6	I can understand nutritionists’ recommendations about health and nutritional requirements that are appropriate for children with disabilities.	3.90 (0.99)
Q8	I can understand information and recommendations about proper nutrition for children with disabilities in the media (e.g., TV, Internet, radio, etc.)	3.96 (0.78)
Q10	I know how different vegetables are cultivated and grown.	3.46 (1.02)
Q16	I encourage children with disability to eat a variety of vegetables (e.g., lettuce, cabbage, tomatoes, carrots, etc.), every day.	4.36 (0.72)
Q19	If I have any questions about food and nutrition issues related to children with disabilities, I’m able to get information and advice from others	3.92 (0.83)
	Functional	4.25 (0.50)
Q13	I know that excessive consumption of food such as salami and sausage that are high in fat may cause obesity among children with disabilities	4.45 (0.65)
Q15	I know that reading of production and expiration date on food package is important for health of children with disabilities	4.51 (0.56)
Q17	I share the nutritional issues that I obtain from various sources with children with disabilities	3.94 (0.78)
Q18	I talk to children with disability about healthy eating.	4.12 (0.81)
Q20	I can advice others to prepare healthy snacks for children with disabilities.	4.23 (0.64)
Q21	I encourage children with disability to have healthy snacks	4.30 (0.60)
Q22	I regularly encourage children with disability to do exercise or walk for 30 to 40 min every day.	4.02 (0.88)
Q23	I can advice others to wash and prepare fruits and vegetables for children with disabilities	4.34 (0.61)
Q24	I encourage children with disabilities to eat fruits every day	4.26 (0.72)
Q25	I make sure children with disabilities eat or they have eaten breakfast every day	4.28 (0.72)
	Interactive	3.53 (0.68)
Q26	I have enough power to stop children with disabilities from eating unhealthy foods (e.g., fast food, pizza, carbonated drinks, etc.)	3.60 (0.94)
Q27	If I go to restaurant or fast food joint with children with disabilities (e.g. pizza, French fries, carbonated drinks, etc.), I’m able to convince him or her to choose healthy foods	3.48 (0.93)
Q28	I can easily convince children with disabilities to say “no” to any unhealthy eating suggestions from friends.	3.40 (0.92)
Q29	If I encounter unhealthy behaviors among children with disabilities at home, school, or in other settings, I’m able to challenge them.	3.68 (0.86)
Q30	If someone prepare unhealthy snacks for children with disabilities (e.g., chips, fruit roll-ups, corn snacks, etc.), I do not allow them to eat in school	3.50 (0.89)
Q34	When I go shopping with children with disabilities, I buy foods with standardized labeling	3.52 (0.83)
	Food choice	3.87 (0.57)
Q31	If parents of children with disabilities were overweight and eating a high fat diet, I would tell them to change their eating habits.	3.81 (0.85)
Q33	When I go shopping with children with disabilities, I buy foods that are certified as healthy	3.89 (0.77)
Q37	When I go shopping with my children with disabilities, I will buy foods that are stored appropriately or kept refrigerated	4.09 (0.74)
Q38	I make sure children with disabilities eat food from all the food groups every day	3.88 (0.84)
Q39	I encourage children with disabilities to try new foods that I’ve never eaten	3.77 (0.89)
Q40	I encourage children with disabilities to try new vegetables that I’ve never eaten	3.93 (0.80)
Q41	I can buy healthy food for children with disabilities from the school cafeteria, depending on the money available.	3.70 (0.80)

M = Mean; SD = Standard deviation

We performed a multigroup moderation analysis to determine the influence of participant type on cognition and understanding using three models. First, we used participant type as a mediator to assess its overall influence on cognition and skills, and found the fit indices to be appropriate (see Table 3, Model 5). Overall, the participant type had both total and indirect effects on the relationship between the independent and dependent variables.

**Fig. 3:**
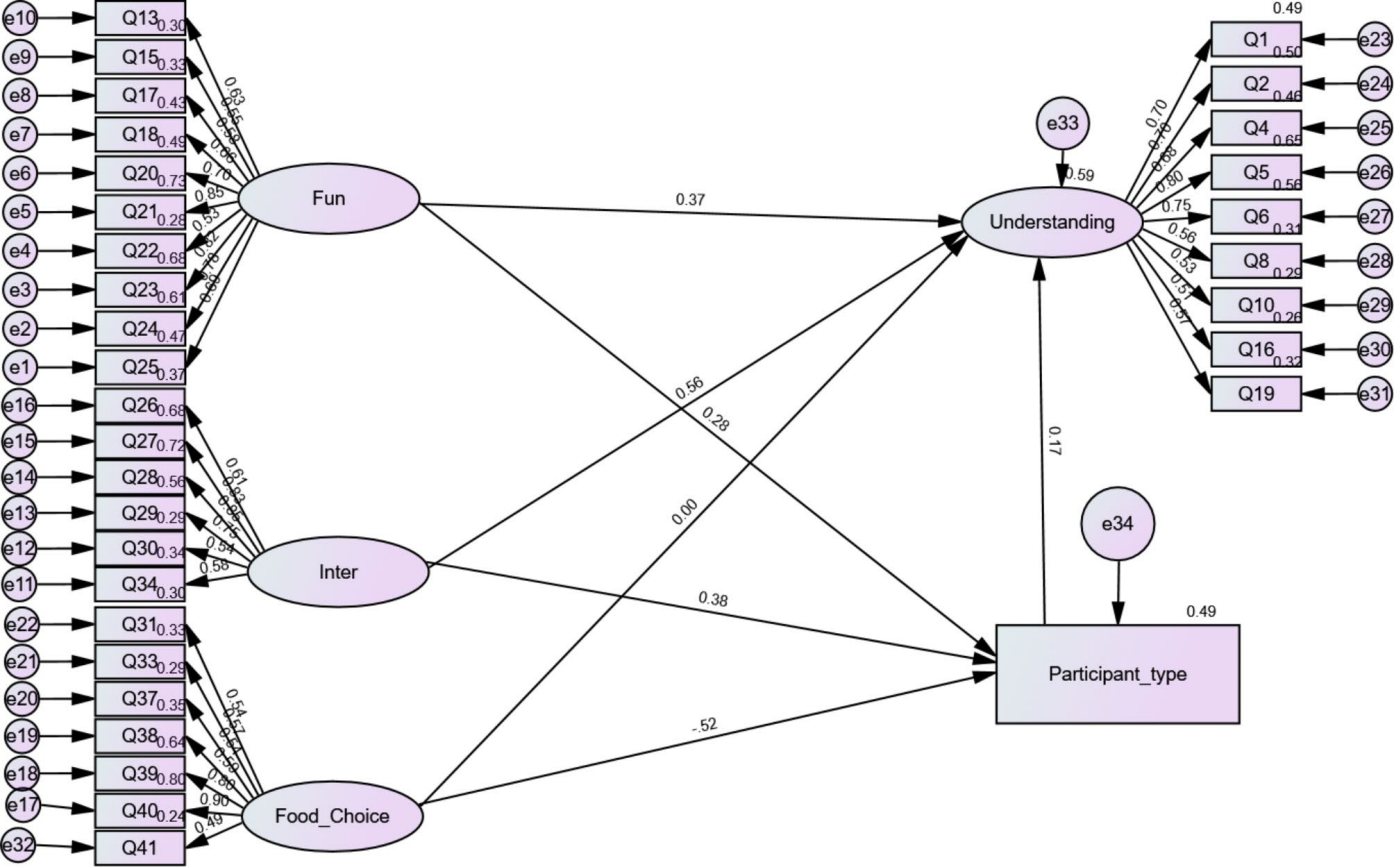
Multigroup moderation analysis results

Individually, participant type affected only interactive (b = 0.27, p = .005) and functional (b = 0.35, p = .002), with a higher standardised direct effect for interactive (b = 0.28, p = .004) but a lower effect for functional (b = 0.19, p = .004). The indirect effect of participant type was also significant for the relationship between functional (b = 0.007, p = .03), interactive (b = 0.010, p = .02), and understanding, and the standardised indirect effect was higher for functional (b = 0.008, p = .03) and lower for interactive (b = 0.007, p = .02), in contrast to the direct effects. Although there was no direct effect of food choice on understanding (b = − 31, p = .35), we found an indirect effect of participant type (b = − 0.21, p = .040) on the influence of food choice on understanding (see Fig. 3).

We also performed a mediation analysis to determine whether being a teacher (n = 78) or a parent (n = 71) individually had an interactive effect on the outcome variable. We created two groups and observed their separate influence on the independent and dependent variables. The fit indices were as follows: chi-square = 2.04, TLI = 0.65, CFI = 0.68, RMSEA = 0.08, and RMSR = 0.12 (see Table 3, Model 6). However, being a parent indirectly mediated the relationship between understanding and interaction (b = 0.63, p = .001); its mediation effects for food choice, functionality, and understanding were insignificant. Second, being a teacher indirectly mediated the relationships between function and understanding (b = 0.53, p = .01) and between interaction and understanding (b = 0.45, p = .02).

## Discussion

In this study, we attempted to explore the awareness of key stakeholders regarding the nutritional needs of children with disabilities in the UAE, guided by Nutbeam’s [40, 41] health literacy model, which emphasises the relevance of cognitive and practical skills in the promotion of individuals’ well-being. The FNLIT [47] seems to be the most comprehensive scale developed based on Nutbeam’s model to assess knowledge of nutrition, and the processes involved in developing the FNLIT were comprehensive and theoretically sound.

In adapting the FNLIT to study knowledge of food nutrition for parents and teachers of children with disabilities in the context of UAE, we found that the two-domain structure (cognitive and skills) was supported; however, the cognitive domain had one subscale, and there were three subscales for skills. This was different from the initial six-factor structure, which comprised two for cognitive skills and four for skills. Nevertheless, we found the revised model to be valid and reliable for studying knowledge of nutrition in the UAE. The EFA supported the factor structure and its appropriateness, and CFA met the assumptions underlying the EFA and appropriate thresholds.

Furthermore, tests of convergent and discriminant validity and Cronbach’s alpha supported the reliability of the revised FNLIT in the UAE context. We argue that the four-subscale structure is valid and measures what it is supposed to measure concerning stakeholders’ understanding of the food and nutritional needs of children with disabilities. Most importantly, the revised scale maintained the hierarchical structure proposed by Nutbeam [40–42] in the conception of the health literacy model.

Hypothesis I is supported by the findings of this study. In particular, there was an apparent relationship between cognition and practical skills, as indicated by the medium-to-large correlations between the subscales. The findings also connote that understanding, interaction, functional, and food choices may depend on one another. This appears to confirm the argument [40, 41] that the two concepts (cognition and practical skills) might be vital for enhancing access to appropriate nutrition for children with disabilities in the UAE. Indeed, knowledge should not be the focus of policymakers; instead, equipping implementors such as teachers and parents with practical skills to monitor the eating habits of children with disabilities could be necessary as well.

There are discussions in the literature concerning poor eating habits and unhealthy lifestyles among children with disabilities [16, 33–36], but it is important to state that they might be unable to make decisions or indicate their preferences. Adults in their lives, such as their parents and teachers, need training to promote better eating habits among them, and to better prepare teachers and parents in the UAE, policymakers could consider training that aims to build confidence in initiating conversations, applying knowledge, and choosing the right food them.

Although there was a correlation between all subscales, we observed a large relationship between two practical factors: functional and food choice. Indeed, Nutbeam [40] discussed health literacy as an asset with many potential influences on society. In this study, we found that food choice and functional skills depended on each other; as functional skills increased, food choice increased as well, and the inverse was true. For example, as parents and teachers gain a better understanding of their functional roles, they are able to select appropriate food and nutrition for children with disabilities. This is expected because the more people know their responsibilities, the better positioned they are to apply their knowledge in real life. In the same way, there is an expectation that when teachers and parents come to terms with core functional duties, they will step up to support children with disabilities in making the right decisions on eating habits. However, the mean score showed that teachers and parents might know their functional roles, but are unsure about helping children choose food. This suggests the need for more attention to developing the skills of teachers and parents in choosing or guiding children to choose the right food for optimal growth.

Hypothesis II is supported by the findings of this study. In particular, two practical skills, functional and interactive, were combined to predict parents’ and teachers’ understanding of the nutritional needs of children with disabilities. Once again, this finding has provided some support to Nutbeam’s literacy model in arguing for the relevance of both knowledge and practical skills. It is prudent to state here that previous studies have reported the susceptibility of children with disabilities to poor eating habits [16, 34–36]. In the context of UAE, it is apparent that if teachers and parents are trained to understand the nutritional needs of children with disabilities, they can perform functional and interactive roles in applying nutritional knowledge, as well as educating others about food and nutrition. This is refreshing in the sense that they should be able to transfer what they have learned to others and support children with disabilities to eat well, which will go a long way towards helping their development. Nevertheless, the inability of food choice to predict understanding might require further discussion to understand what could be done to enable teachers and parents to help children with disabilities eat the right nutritious food.

These findings partially support Hypothesis III. Specifically, being a parent or teacher moderates the relationship between cognition and practical nutritional skills. The findings showed that participation had both direct and indirect effects on the relationships between interactive, functional, and understanding. Moreover, participant type had an indirect effect on the relationship between food choice and the understanding of food and nutritional needs of children with disabilities. This finding suggests that policymakers might be unable to increase access to more nutritious foods for children with disabilities without the involvement of teachers and parents. It is undeniable that these two populations are stakeholders with the most significant engagement with children with disabilities, making them important stakeholders in the effort to promote better eating habits among them, yet they face barriers in caring for them in the UAE [52, 53]. This finding calls for engagement between policymakers and these stakeholders to understand their concerns and support them in promoting better eating habits among children with disabilities, for instance, in the form of regular nutrition training. To achieve an inclusive society in the UAE, it is critical to develop the potential of adults in the lives of children with disabilities to expand their knowledge of healthy food choices and access to the right foods for a fulfilling life.

## Study limitations

Due to several limitations, it is not possible to generalise the findings of this study to broader populations. First, we collected the study data online because of COVID-19 restrictions on in-person education, and thus, we did not have direct contact with potential participants; therefore, we could not be certain whether the right participant completed the questionnaire. However, we collected data from recognised institutions, and there is a likelihood that they only provided access to participants who met the inclusion criteria. Second, the participants self-reported their nutritional knowledge and skills, and there was a high likelihood of participant deception in self-assessments. Nevertheless, the study and its objectives were in both Arabic and English, which meant that participants read and answered the questionnaire in their preferred language of proficiency. Overall, this study is the first time the FNLIT has been validated and used to assess nutritional knowledge in a context such as the UAE.

## Conclusion and practical implications

In this study, we attempted to understand parents’ and teachers’ understanding of the food and nutritional needs of children with disabilities. We used Nutbeam’s health literacy model as a framework to test three hypotheses, according to which knowledge and skills are vital to achieving health literacy. In adopting the FNLIT scale, we identified one cognitive subscale (understanding) and three skill subscales (functional, interactive, and food choice) supported by the EFA, CFA, and reliability tests. In this study, we confirmed a relationship between understanding and practical nutritional skills (Hypothesis I), and found that two practical nutritional skills, functional and interactive, combined to predict the understanding of nutritional needs of children with disabilities (Hypothesis II). Furthermore, Hypothesis III was partially supported in that participant type mediated the relationship between functional, interactive, and understanding. The findings demonstrate the centrality of teachers and parents in the effort to promote better eating habits among children with disabilities in the UAE.

The findings of this study have implications for UAE’s policies and practice. First, health policymakers should consider developing the knowledge and practical skills of both parents and teachers, including practical implementation skills. The training could be tailored to suit specific disability groups to help teachers and parents acquire and apply relevant skills. Second, parents and teachers should be trained about their functional roles and the foods required by specific disability groups. This would enable them to help children with specific disabilities access appropriate nutrition and, more broadly, support children with disabilities to develop better eating habits.

## Data Availability

The datasets generated and analysed during the current study are not publicly available due to ethical restrictions but are available from the corresponding author upon reasonable request.

## List of abbreviations

CFA: Confirmatory factor analysis.
CFI: Comparative fit index.
EFA: Exploratory factor analysis.
SRMR: Standard root mean square residual.
UAE: United Arab Emirates.
